# Impact of bariatric surgery on the resolution of obesity hypoventilation syndrome at 1-year follow-up: a retrospective study

**DOI:** 10.5664/jcsm.11750

**Published:** 2025-10-01

**Authors:** Shuai Ma, Wenwen Yu, Chengcan Yang, Yining He, Dongzi Zhu, Fen Gu, Bei Xu, Xiaozhen Xu, Kan Yao, Xiurong Tao, Min Zhu, Bing Wang

**Affiliations:** ^1^ Department of General Surgery, Shanghai Ninth People’s Hospital, Shanghai Jiao Tong University School of Medicine, Shanghai, China; ^2^ Department of Oral and Cranio-Maxillofacial Surgery, Sleep-Disordered Breathing Center, Shanghai Ninth People’s Hospital, Shanghai Jiao Tong University School of Medicine, Shanghai, China; ^3^ Biostatistics Office of Clinical Research Unit, Shanghai Ninth People’s Hospital, Shanghai Jiao Tong University School of Medicine, Shanghai, China; ^4^ Nursing Department, Shanghai Ninth People’s Hospital, Shanghai Jiao Tong University School of Medicine, Shanghai, China

**Keywords:** obesity hypoventilation syndrome, sleep-disordered breathing, metabolic and bariatric surgery, obesity, arterial blood gas, hypercapnia, risk factors

## Abstract

**Study Objectives:**

This study aimed to assess the effectiveness of metabolic and bariatric surgery in patients with obesity comorbid with obesity hypoventilation syndrome (OHS) at 1-year follow-up.

**Methods:**

This retrospective study was conducted between January 2020 and June 2023 at a metabolic and bariatric surgery center in a university-affiliated tertiary hospital in China. Clinical data, including body mass index, arterial blood gas values, portable sleep study results, and anthropometric parameters, were recorded pre- and postoperatively. Correlations between variables and risk factors for OHS resolution were analyzed.

**Results:**

Among 1,134 candidates for metabolic and bariatric surgery, 187 (16.5%) had comorbid OHS; 151 patients with OHS met inclusion criteria and completed the 1-year follow-up visit (body mass index 39.1 ± 6.8 kg/m^2^ with partial pressure of carbon dioxide in arterial blood [PaCO_2_] 48.6 ± 3.0 mmHg). At 1-year follow-up, body mass index decreased to 29.0 ± 6.0 kg/m^2^ (*P* < .001) and PaCO_2_ dropped to 43.8 ± 5.5 mmHg (*P* < .001). Resolution of OHS, defined as awake PaCO_2_ < 45 mmHg with discontinuation of positive airway pressure therapy for a minimum of 6 months before obtaining the arterial blood gas at the 12-month visit, was achieved in 105 (69.5%) of the patients. Nonlinear analysis indicated that PaCO_2_ did not significantly decrease until the percentage of total weight loss exceeded approximately 20%. A larger reduction in waist circumference was associated with a greater reduction in PaCO_2_, particularly when waist circumference reached less than 25 cm. Beyond this point, ΔPaCO_2_ reached a plateau. In multivariate analysis, a larger preoperative waist circumference (odds ratio: 1.046, 95% confidence interval: 1.031–1.118, *P =* .025) and arterial blood gas pH < 7.35 (odds ratio: 3.921, 95% confidence interval: 2.305–9.140, *P* < .001) were associated with lack of resolution of OHS, and a larger percentage of total weight loss after bariatric surgery (odds ratio: 0.917, 95% confidence interval: 0.846–0.965, *P =* .001) was independently associated with OHS resolution.

**Conclusions:**

Metabolic and bariatric surgery is an effective treatment for OHS. Achieving a sufficient percentage of total weight loss is critical for the resolution of OHS.

**Citation:**

Ma S, Yu W, Yang C, et al. Impact of bariatric surgery on the resolution of obesity hypoventilation syndrome at 1-year follow-up: a retrospective study. *J Clin Sleep Med.* 2025;21(10):1665–1678.

**BRIEF SUMMARY**

**Current Knowledge/Study Rationale:** This study aimed to assess the impact of metabolic and bariatric surgery on patients with obesity hypoventilation syndrome. Given the limited effectiveness of surgical interventions in improving obesity hypoventilation syndrome, this study investigated how metabolic and bariatric surgery might facilitate reductions in weight and improvements in arterial blood gases.

**Study Impact:** Current guidelines, such as those from the American Thoracic Society, recommend a weight loss target of 25–30% in patients with obesity hypoventilation syndrome, but these recommendations are largely based on expert consensus rather than direct clinical evidence. Our study provides the first evidence-based quantitative target, while also highlighting that central obesity and preoperative pH level are independently associated with the resolution of obesity hypoventilation syndrome.

## INTRODUCTION

Obesity has emerged as a critical global health issue, contributing to significant morbidity and mortality. According to the World Health Organization,^1^ more than 1.9 billion adults aged 18 years or older are classified as overweight, with 650 million falling into the obese category. In China, the prevalence of overweight and obesity has escalated dramatically, with 34.8% of adults classified as overweight and 14.1% as obese.^2^ The burden of obesity, measured in disability-adjusted life years, has surged 2.7-fold, rising from 48 million in 1990 to 129 million in 2021, highlighting the growing impact of metabolic diseases nationwide.^3^

Among the general adult population, the prevalence of obstructive sleep apnea (OSA), defined by an apnea-hypopnea index (AHI) measurement threshold of ≥ 5 events/h, ranges from 9–38%.^4^^,^^5^ A conservative estimation suggests that approximately half of those with this degree of obesity have some degree of OSA, leading to an estimated prevalence of obesity hypoventilation syndrome (OHS) of around 0.4%^6^ within the general population. Moreover, the incidence of OHS has been observed to rise in conjunction with increasing obesity rates,^7^^,^^8^ reaching 27% in individuals with a body mass index (BMI) over 40 kg/m^2^ and climbing as high as 50% in those with a BMI exceeding 50 kg/m^2^.^8^

The primary approach to managing OHS involves the use of positive airway pressure (PAP) therapy during sleep to alleviate sleep-disordered breathing, alongside efforts to reduce other risks by addressing severe obesity.^8^^,^^9^ However, despite the potential benefits of consistent PAP therapy usage, research indicates that the cardiometabolic risk factors associated with severe obesity may persist.^6^^,^^10^^,^^11^ Lifestyle interventions have shown limited long-term success in managing OHS, because weight gain recurrence often undermines initial improvements from diet and exercise programs.^12^^,^^13^ The American Thoracic Society guidelines^10^ recommend a weight loss goal of 25–30% for patients with OHS. Metabolic and bariatric surgery (MBS) has proven more effective at achieving and sustaining substantial weight loss, meeting the American Thoracic Society recommendations. However, although the epidemiology of OHS in preoperative MBS cohorts has been documented, few studies have specifically examined the impact of bariatric surgery on OHS treatment outcomes.^10^^,^^14^ Therefore, this retrospective study aimed to evaluate the effects of MBS on OHS and to identify associated risk factors influencing treatment outcomes.

## METHODS

### Study design

This retrospective cohort study included patients who underwent MBS between January 2020 and June 2023 at Shanghai Ninth People’s Hospital, Shanghai Jiao Tong University School of Medicine, Shanghai, China. The protocol was approval by the institutional review board.

### Definitions

OHS was defined as a combination of obesity (BMI ≥ 30 kg/m^2^) and daytime awake hypercapnia (partial pressure of carbon dioxide in arterial blood [PaCO_2_] ≥ 45 mmHg), after excluding other causes of hypoventilation.^10^ Postoperative resolution of OHS was defined^10^ as awake PaCO_2_ < 45 mmHg after discontinuation of PAP therapy for a minimum of 6 months before obtaining the arterial blood gas (ABG) values at the 12-month visit.

### Inclusion and exclusion criteria

The inclusion criteria were (1) patients who underwent MBS based on surgical indications^9^ and (2) diagnosis of OHS.^10^ The exclusion criteria were (1) history of craniomaxillofacial, oral, or epigastric diseases and/or surgeries; (2) history of malignant tumors; and (3) failure to follow up at 1 year.

### Data collection

Data were collected at the preoperative evaluation (on the day of admission) and at 1-year postoperative follow-up. Clinical characteristics including sex, age, height, weight, BMI, surgical procedures, ABG, portable sleep study results, anthropometric parameters (neck, chest, waist, and hip circumference; see supplemental material), pulmonary function tests, B-type natriuretic peptide, echocardiography, red blood cell count, triglycerides, total cholesterol, fasting plasma glucose, glycated hemoglobin, and blood pressure were reviewed based on the medical records. ABG was performed in accordance with the American Association for Respiratory Care Clinical Practice guidelines.^15^ The diagnosis of OHS was identified using the *International Classification of Diseases, 10th revision*, Clinical Modification code E66.2, which is assigned during multidisciplinary team (MDT) evaluations and recorded in the electronic medical record.

### Portable sleep study

The sleep study adopted in this study was an attended cardiorespiratory sleep study. The device (Alice NightOne; Philips Respironics, Murrysville, Pennsylvania) used as a type III portable sleep monitor in this study has 3 sensors (a chest effort belt, a nasal pressure cannula, and an oximeter). Sleep staging and arousals were not assessed in this study. Each sleep study was performed in accordance with American Academy of Sleep Medicine guidelines.^16^ Studies were scored by experienced registered polysomnography technicians according to standard practices and then reviewed and interpreted by an experienced sleep specialist. Because the sleep testing device used in the study did not have an oronasal thermistor, nasal pressure transducer signals were used to score apneas. An obstructive apnea was scored when there was a drop in the peak flow signal excursions by ≥ 90% from the nasal pressure transducer for ≥ 10 seconds and associated with the presence of respiratory effort. Central apnea was defined as absent respiratory effort for at least 10 seconds throughout the entire period of absence of airflow. Hypopnea was scored when the peak signal excursions dropped by ≥ 30% from the nasal pressure transducer for ≥ 10 seconds and was associated with ≥ 3% oxygen desaturation. The AHI was calculated as the total number of apneas and hypopneas per hour during the total recording time. The oxygen desaturation index was calculated as the total number of 3% or greater oxygen desaturations per hour during the total recording time. Based on the AHI, OSA was classified as absent (< 5), mild (≥ 5–14.9), moderate (≥ 15–29.9), or severe (≥ 30). Other data derived from sleep studies, including mean oxygen saturation, nadir oxygen saturation, longest apnea time, and percentage of oxygen saturation less than 90% in total recording time were also collected. Sleep studies were performed on the night of initial hospital admission for surgery and on the night of hospital admission during the follow-up period, with results reviewed by experienced sleep specialists. PAP therapy was discontinued for 2 nights prior to the sleep studies.

### Echocardiography

Echocardiographic data were collected retrospectively from reports available in the hospital’s electronic medical record system. These reports were primarily used for preoperative evaluations as part of the multidisciplinary assessment. Right ventricular systolic pressure was estimated using the tricuspid regurgitation jet. Because echocardiography does not directly measure pulmonary arterial pressure, right ventricular systolic pressure and pulmonary artery systolic pressure are considered interchangeable in the absence of pulmonic valve defects. Due to the retrospective nature of this study, tricuspid regurgitation–derived right ventricular systolic pressure values were recorded directly from available echocardiography reports, and not all patients had measurable tricuspid regurgitation jet signals. Pulmonary artery diameter was measured at approximately 1 cm above the pulmonary valve using a manual measurement technique. These measurements were obtained from routine echocardiographic reports documented in the hospital’s electronic medical records. Left ventricular ejection fraction values were obtained from echocardiography reports to assess global left ventricular systolic function. Given the retrospective study design, not all patients had complete echocardiographic data, because the examinations were conducted based on clinical indications and institutional protocols.

### Perioperative management and follow-up

Perioperative management and follow-up were conducted per standard clinical practice.^17^^,^^18^ An MDT managed therapeutic strategies for patients with confirmed awake hypercapnia preoperatively and postoperatively, including PAP therapy.^8^^,^^19^ Detailed standard operating procedures for perioperative management of OHS are available in the supplemental material. Patients were scheduled for postoperative follow-up visits at 1, 3, 6, and 12 months and underwent ABG at each postoperative visit. To minimize short-term effects of PAP on PaCO_2_ measurements, patients were instructed to stop using PAP for at least 2 nights prior to each ABG test.

### Surgical procedures

Laparoscopic sleeve gastrectomy, laparoscopic Roux-en-Y gastric bypass, and single anastomosis duodeno-ileostomy with sleeve gastrectomy were performed according to the latest guidelines.^9^^,^^17^^,^^18^^,^^20^

### Statistical analysis

The comparison of continuous variables between 2 groups was performed using an independent sample *t* test or a paired *t* test, and the comparison among 3 groups was conducted using analysis of variance. Categorical variables were analyzed using the χ^2^ test or Fisher’s exact test. Correlations were identified using heatmaps and restricted cubic splines. Binary logistic regression was applied for univariate and multivariate analyses to assess risk factors. To assess the homogeneity of the cohorts for bariatric surgery, we performed a homogeneity analysis comparing baseline characteristics across OHS and non-OHS groups. Variables with *P* < .05 in univariate analysis were included in multivariate analysis. Results were considered statistically significant at *P* < .05. Statistical analyses were performed using IBM SPSS Statistics software version 25 for Mac (IBM Corp., Armonk, New York), R software version 4.3.0 for Mac (R Foundation for Statistical Computing, Vienna, Austria), and Prism 8 software for Mac (GraphPad LLC, San Diego, California).

## RESULTS

### Baseline characteristics

**Figure 1:**
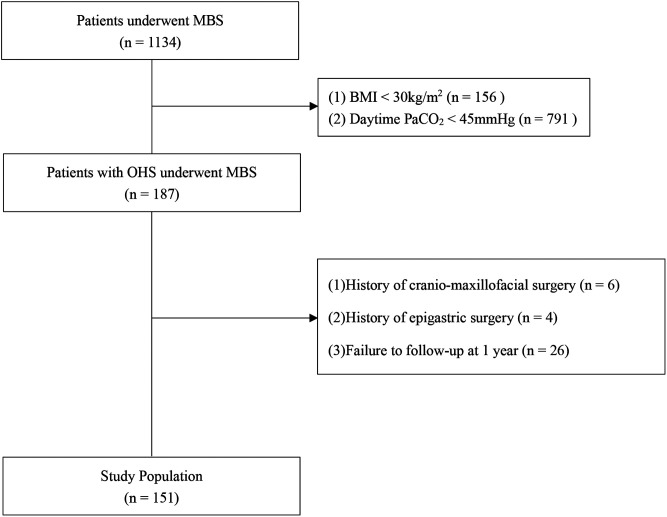
Flowchart illustrating the study approach. BMI = body mass index, MBS = metabolic and bariatric surgery, OHS = obesity hypoventilation syndrome, PaCO_2_ = partial pressure of carbon dioxide in arterial blood.

A total of 1,134 MBS candidates (**Figure 1**) were recruited at our center from January 2020 to June 2023. Among these, 16.5% were diagnosed with OHS, of whom 81.3% had available follow-up data at the 1-year mark after MBS. Ultimately, 151 eligible patients with an average BMI of 39.1 ± 6.8 kg/m^2^ were enrolled in this study (**Table 1**). The mean age was 32.6 ± 10.8 years, and 60.9% of the cohort were male. The distribution of surgical procedures included 58.3% undergoing laparoscopic sleeve gastrectomy, 34.4% undergoing laparoscopic Roux-en-Y gastric bypass, and 7.3% undergoing single anastomosis duodeno-ileostomy with sleeve gastrectomy (**Table 1**).

**Table 1:** Baseline of patients with and without OHS.

	OHS (n = 151)	Non-OHS (n = 791)			*P* ^a^
		Total (n = 791)	Eucapnic No/Mild OSA (n = 354)	Eucapnic Moderate/Severe OSA (n = 437)	
Age (years)	32.6 ± 10.8	32.4 ± 7.9	32.8 ± 8.3	31.9 ± 7.4	.121
Male/female	92/59	384/407	131/223	253/184	**<.001**
BMI (kg/m^2^)	39.1 ± 6.8	38.0 ± 6.2	36.9 ± 4.7	40.4 ± 7.1	**.018**
LSG/LRYGB/SADI-S	88/52/11	493/280/18	216/135/3	277/145/15	.089
%TWL	25.2 ± 12.3	25.7 ± 11.2	24.9 ± 10.5	26.1 ± 13.7	.391
RBC count (×10^12^/l)	4.81 ± 0.55	4.62 ± 0.77	4.34 ± 0.43	4.89 ± 0.82	.312
BNP (pg/ml)	13.6 ± 16.9	12.5 ± 15.2	11.8 ± 14.6	13.1 ± 15.9	.284
RVSP (mmHg)^b^	31.5 ± 5.1	29.3 ± 4.7	28.4 ± 4.5	30.5 ± 5.2	.491
Pulmonary artery diameter (mm)^b^	24.4 ± 1.7	24.3 ± 1.6	24.2 ± 1.5	25.1 ± 1.9	.389
LVEF (%)	64.0 ± 3.1	62.1 ± 2.9	64.6 ± 2.1	60.8 ± 3.7	.418
FVC (% predicted)	86.7 ± 10.4	90.2 ± 10.5	93.5 ± 10.2	88.6 ± 11.4	.332
FEV_1_ (% predicted)	81.7 ± 11.5	84.8 ± 11.3	86.5 ± 10.8	81.3 ± 12.2	.267
FEV_1_/FVC (%)	82.1 ± 4.9	85.0 ± 4.9	87.1 ± 4.8	82.0 ± 5.2	.375
ABG					
pH	7.33 ± 0.03	7.38 ± 0.03	7.39 ± 0.02	7.38 ± 0.03	**<.001**
PaCO_2_ (mmHg)	48.6 ± 3.0	39.8 ± 2.8	37.5 ± 2.3	40.6 ± 2.9	**<.001**
PaO_2_ (mmHg)	90.1 ± 18.8	89.2 ± 16.1	93.5 ± 15.7	87.3 ± 20.6	**.028**
HCO_3_^−^ (mmol/l)	25.8 ± 2.2	24.2 ± 2.0	23.1 ± 1.9	24.7 ± 2.6	**<.001**
cLac (mmol/l)	2.6 ± 0.9	1.8 ± 0.7	1.6 ± 0.3	2.0 ± 1.1	**.034**
Portable sleep study					
AHI (events/h)	45.1 ± 35.5	31.3 ± 26.4	8.7 ± 3.2	44.7 ± 31.2	**<.001**
ODI (/h)	45.5 ± 34.7	27.0 ± 19.2	6.8 ± 3.0	48.4 ± 22.5	**<.001**
LAT (seconds)	57.3 ± 24.9	56.8 ± 24.6	49.3 ± 22.9	63.1 ± 25.0	.069
SIT90 (%)	12.3 ± 17.1	9.9 ± 14.6	4.3 ± 5.5	14.7 ± 18.1	**<.001**
Mean SpO_2_ (%)	90.9 ± 5.7	93.1 ± 4.6	94.9 ± 3.9	91.3 ± 5.1	**<.001**
Nadir SpO_2_ (%)	70.7 ± 14.8	73.6 ± 13.9	81.3 ± 10.7	69.3 ± 15.5	**<.001**

According to AHI, OSA could be assigned to 1 of 4 severity categories: absent (< 5), mild (≥ 5–14.9), moderate (≥ 15–29.9), or severe (≥ 30). Statistically significant values are indicated in bold. ^a^One-way analysis of variance for continuous variables among the 3 groups: the OHS group, the eucapnic no/mild OSA group, and the eucapnic moderate/severe OSA group. Statistically significant values are indicated in bold. ^b^Groups with data completeness rates below 100%, with pulmonary arterial pressure at 83.4% and pulmonary artery diameter at 93.4%. AHI = apnea-hypopnea index, BMI = body mass index, BNP = brain natriuretic peptide, cLac = concentration of lactic acid, FEV_1_ = forced expiratory volume in 1 second, FVC = forced vital capacity, HCO_3_^−^ = calculated bicarbonate from the arterial blood gas, LAT = longest apnea time, LRYGB = laparoscopic Roux-en-Y gastric bypass, LSG = laparoscopic sleeve gastrectomy, LVEF = left ventricle ejection fraction, MBS = metabolic and bariatric surgery, ODI = oxygen desaturation index, OHS = obesity hypoventilation syndrome, OSA = obstructive sleep apnea, PaCO_2_ = partial pressure of carbon dioxide in arterial blood, PaO_2_ = partial pressure of arterial oxygen, RBC = red blood cell, RVSP = right ventricular systolic pressure, SADI-S = single anastomosis duodeno-ileal bypass with sleeve gastrectomy, SIT90 = the percentage of oxygen saturation less than 90% in total recording time, SpO_2_ = saturation of percutaneous oxygen.

We conducted a homogeneity analysis between OHS cases and non-OHS cases (**Table 1**). The results showed that BMI in the OHS group and the eucapnic moderate/severe OSA group was higher than those in the eucapnic no/mild OSA group. Across all groups, the distribution of surgical procedures and percentage of total weight loss (%TWL) was not significantly different.

Following the preoperative work-up process, all patients diagnosed with OHS (151/151) received preoperative PAP therapy under MDT supervision while on the surgical ward. None of these patients had been previously diagnosed with OHS or had received PAP therapy at home prior to admission. Patients were instructed to use the noninvasive ventilation for more than 4 hours each night, with real-time monitoring by on-duty medical staff. Adherence was verified through nursing documentation and overnight shift reports. If the ventilator showed no alarm activations or abnormalities, and if nurses confirmed—through routine checks every 2 hours—that the patient was using the device appropriately, the patient was considered to have met the nightly usage requirement. Since all PAP therapy was administered during hospitalization under direct supervision, all 151 patients with OHS in the cohort met the PAP adherence criteria. In addition, to optimize therapeutic efficacy, patients underwent compulsory daytime PAP acclimatization sessions during hospitalization. The mean duration of preoperative PAP therapy while the patients were hospitalized before elective surgery was 6.3 ± 1.8 days (median of 5 days, minimum and maximum of 3 and 18 days). All 151 patients with OHS were discharged from the hospital with instructions to continue home PAP therapy, as recommended by the MDT. However, objective PAP adherence data was not available.

### Safety of MBS for patients with OHS

As shown in **Table 2**, the perioperative safety of patients in the OHS and non-OHS groups was comparable. None of the enrolled patients experienced circulatory or respiratory failure. No reintubation, reoperation, or readmission occurred during the perioperative period. Notably, no cases in the eucapnic no/mild OSA group required admission to the intensive care unit. Meanwhile, the intensive care unit admissions in the OHS group were primarily precautionary clinical measures. Patients from the OHS group typically stayed in the intensive care unit for approximately 1 day postoperatively for observation before being transferred to the general ward.

**Table 2:** Surgical safety between OHS cohort and non-OHS cohort.

	OHS (n = 151)	Non-OHS (n = 791)			*P*
		Total (n = 791)	Eucapnic No/Mild OSA (n = 354)	Eucapnic Moderate/Severe OSA (n = 437)	
Operative time (minutes)	117.4 ± 8.5	109.8 ± 12.2	110.0 ± 10.6	109.5 ± 13.1	.565^a^
Blood loss (ml)	15.3 ± 5.1	19.0 ± 10.9	18.6 ± 9.5	19.4 ± 12.0	.372^a^
Postoperative LOS (days)	3.4 ± 1.6	3.2 ± 1.7	3.1 ± 1.4	3.2 ± 2.0	.148^a^
ICU admission, n (%)	9 (6.0%)	37 (4.7%)	0 (0.0%)	37 (8.5%)	.416^b^
ICU stay (days)	1.56 ± 0.53	1.62 ± 0.73	—	1.62 ± 0.73	.789^c^
Emergent intubation, n (%)	0 (0.0%)	0 (0.0%)	0 (0.0%)	0 (0.0%)	—
Short-term complication, n (%)					.132^d^
Hemorrhage	3 (2.0%)	6 (0.8%)	1 (0.3%)	5 (1.1%)	—
Leakage	0 (0.0%)	0 (0.0%)	0 (0.0%)	0 (0.0%)	—
Pneumonia	2 (1.3%)	8 (1.0%)	2 (0.6%)	6 (1.4%)	—
Obstruction	0 (0.0%)	3 (0.4%)	0 (0.0%)	3 (0.7%)	—

^a^One-way analysis of variance for continuous variables among the 3 groups: the OHS group, the eucapnic no/mild OSA group, and the eucapnic moderate/severe OSA group. Statistically significant values are indicated in bold. ^b^χ^2^ test for categorical data between the OHS group and the eucapnic moderate/severe OSA group. ^c^Independent sample *t* test for continuous variables between the OHS group and the eucapnic moderate/severe OSA group. ^d^Fisher’s exact test for categorical variables in the OHS group, the eucapnic no/mild OSA group, and the eucapnic moderate/severe OSA group. ICU = intensive care unit, LOS = length of hospital stay, OHS = obesity hypoventilation syndrome, OSA = obstructive sleep apnea.

### Effectiveness of MBS on OHS and obesity-related comorbidity resolution

**Figure 2:**
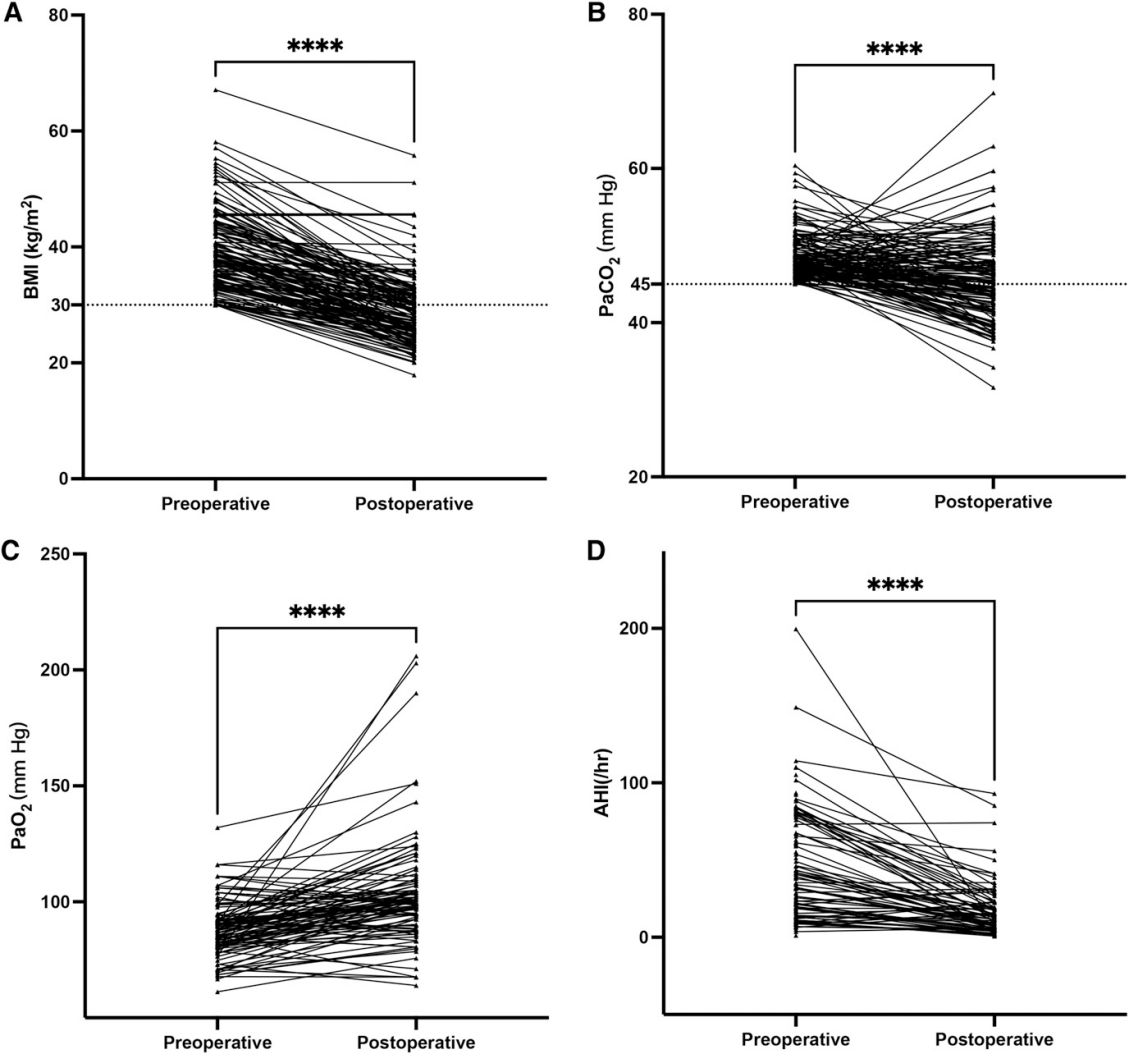
Effectiveness of MBS and OHS resolution. Paired *t* test for effectiveness of MBS for OHS (n = 151). **(A)** BMI. **(B)** PaCO_2_. **(C)** PaO_2_. **(D)** AHI. ****P < .0001. AHI = apnea-hypopnea index, BMI = body mass index, MBS = metabolic and bariatric surgery, OHS = obesity hypoventilation syndrome, PaCO_2_ = partial pressure of carbon dioxide in arterial blood, PaO_2_ = partial pressure of arterial oxygen.

At 1-year follow-up, OHS resolution was observed in 105 out of 151 (69.5%) patients (**Table 3**, **Table 4**, and **Figure 2**). Of these 105 patients, PAP therapy was discontinued due to resolution of hypercapnia at the 3-month visit in 89 patients and at the 6-month visit in 16 patients. Therefore, these 105 patients had sustained eucapnia at the 12-month visit despite discontinuation of PAP therapy for 6–9 months. Among all 151 patients with OHS (**Table 3**), BMI was significantly reduced from 39.1 ± 6.8 kg/m^2^ to 29.0 ± 6.0 kg/m^2^ (*P* < .001), with a %TWL of 25.2% ± 12.3%. Additionally, there was a statistically significant improvement in pH (7.33 ± 0.03 vs 7.35 ± 0.04, *P =* .001) and PaO_2_ (91.0 ± 18.8 vs 98.2 ± 26.6 mmHg, *P =* .005) and a decrease in PaCO_2_ (48.6 ± 3.0 vs 43.8 ± 5.5mmol/L, *P* < .001) postsurgery.

**Table 3:** Effectiveness of MBS in patients with and without OHS.

	OHS (n = 151)		*P* ^a^	Non-OHS Total (n = 791)		*P* ^a^	Eucapnic No/Mild OSA (n = 354)		*P* ^a^	Eucapnic Moderate/Severe OSA (n = 437)		*P* ^a^	*P* ^b^
	Preoperative	Postoperative		Preoperative	Postoperative		Preoperative	Postoperative		Preoperative	Postoperative		
Anthropometric parameters													
Weight (kg)	114.1 ± 25.0	84.5 ± 21.1	**<.001**	111.5 ± 28.4	83.8 ± 22.3	**<.001**	103.4 ± 19.8	78.3 ± 16.9	**<.001**	120.8 ± 33.6	89.5 ± 25.8	**<.001**	.182
BMI (kg/m^2^)	39.1 ± 6.8	29.0 ± 6.0	**<.001**	38.0 ± 6.2	28.7 ± 5.5	**<.001**	36.9 ± 4.7	27.4 ± 4.3	**<.001**	40.4 ± 7.1	30.0 ± 5.7	**<.001**	.273
Circumference													
Neck (cm)	44.2 ± 5.2	38.9 ± 4.8	**<.001**	43.5 ± 4.8	38.5 ± 4.5	**<.001**	42.1 ± 4.3	37.6 ± 4.0	**<.001**	44.8 ± 5.1	39.4 ± 4.7	**<.001**	.314
Chest (cm)	122.2 ± 14.1	106.6 ± 16.4	**<.001**	120.1 ± 13.8	105.2 ± 14.9	**<.001**	118.0 ± 12.5	103.8 ± 14.2	**<.001**	121.7 ± 13.9	106.0 ± 15.5	**<.001**	.299
Waist (cm)	121.8 ± 16.3	100.2 ± 16.8	**<.001**	119.6 ± 15.7	98.7 ± 16.2	**<.001**	116.9 ± 14.1	95.6 ± 14.9	**<.001**	121.0 ± 15.8	99.3 ± 16.5	**<.001**	.871
Hip (cm)	124.3 ± 14.6	107.8 ± 12.9	**<.001**	122.5 ± 14.3	106.2 ± 12.5	**<.001**	120.3 ± 13.7	104.4 ± 12.1	**<.001**	123.8 ± 14.7	107.5 ± 12.7	**<.001**	.306
Neck/chest	0.36 ± 0.03	0.37 ± 0.04	.398	0.35 ± 0.03	0.36 ± 0.03	.192	0.34 ± 0.03	0.34 ± 0.04	.498	0.36 ± 0.04	0.36 ± 0.03	.248	.283
Neck/waist	0.36 ± 0.04	0.39 ± 0.05	**<.001**	0.36 ± 0.04	0.36 ± 0.05	.178	0.35 ± 0.04	0.34 ± 0.05	.511	0.37 ± 0.04	0.37 ± 0.05	.289	.325
Neck/hip	0.35 ± 0.03	0.36 ± 0.04	.050	0.35 ± 0.03	0.35 ± 0.03	.210	0.34 ± 0.03	0.33 ± 0.03	.530	0.37 ± 0.05	0.36 ± 0.03	.312	.401
Chest/waist	1.01 ± 0.06	1.07 ± 0.09	**.001**	1.01 ± 0.07	1.07 ± 0.08	**.003**	1.02 ± 0.07	1.02 ± 0.06	.556	1.00 ± 0.06	1.08 ± 0.08	**<.001**	.388
Chest/hip	0.98 ± 0.07	0.99 ± 0.06	.210	0.97 ± 0.06	0.98 ± 0.08	.447	0.97 ± 0.06	0.98 ± 0.07	.812	0.96 ± 0.07	0.97 ± 0.08	.696	.268
Waist/hip	0.98 ± 0.07	0.93 ± 0.09	**<.001**	0.97 ± 0.07	0.93 ± 0.08	**.002**	0.96 ± 0.06	0.96 ± 0.07	.401	0.98 ± 0.07	0.92 ± 0.08	**.001**	.090
Arterial blood gas													
pH	7.33 ± 0.03	7.35 ± 0.04	**.001**	7.38 ± 0.03	7.39 ± 0.03	.226	7.39 ± 0.02	7.38 ± 0.02	.498	7.38 ± 0.03	7.39 ± 0.03	.312	**<.001**
PaCO_2_ (mm Hg)	48.6 ± 3.0	43.8 ± 5.5	**<.001**	39.8 ± 2.8	39.5 ± 2.7	.180	37.5 ± 2.3	37.3 ± 2.5	.512	40.6 ± 2.9	39.9 ± 2.8	.289	**<.001**
PaO_2_ (mm Hg)	90.1 ± 18.8	98.2 ± 26.6	**.005**	89.2 ± 16.1	94.8 ± 17.9	**.030**	93.5 ± 15.7	94.5 ± 16.0	.556	87.3 ± 20.6	95.1 ± 18.7	**.007**	**.001**
HCO_3_^−^ (mmol/l)	25.8 ± 2.2	25.4 ± 2.7	.125	24.2 ± 2.0	24.1 ± 2.3	.318	23.1 ± 1.9	23.0 ± 2.1	.405	24.7 ± 2.6	24.5 ± 2.5	.268	.531
cLac (mmol/l)	2.6 ± 0.9	2.0 ± 0.7	**<.001**	1.8 ± 0.7	1.7 ± 0.5	.275	1.6 ± 0.3	1.5 ± 0.2	.401	2.0 ± 1.1	1.9 ± 1.0	.388	**.002**
Portable sleep study													
AHI (events/h)	45.1 ± 35.5	17.7 ± 18.1	**<.001**	31.3 ± 26.4	12.5 ± 11.3	**<.001**	8.7 ± 3.2	5.5 ± 2.1	**.014**	44.7 ± 31.2	19.2 ± 15.7	**<.001**	**<.001**
ODI (/h)	45.5 ± 34.7	14.6 ± 15.5	**<.001**	27.0 ± 19.2	10.2 ± 8.6	**<.001**	6.8 ± 3.0	4.9 ± 2.3	**.038**	48.4 ± 22.5	16.5 ± 12.1	**<.001**	**<.001**
LAT (seconds)	57.3 ± 24.9	52.4 ± 24.8	.197	56.8 ± 24.6	54.7 ± 22.1	.211	49.3 ± 22.9	47.8 ± 21.4	.347	63.1 ± 25.0	58.6 ± 24.3	.226	.317
SIT90 (%)	12.3 ± 17.1	2.7 ± 6.4	**<.001**	9.9 ± 14.6	3.3 ± 7.8	**<.001**	4.3 ± 5.5	3.0 ± 3.2	.129	14.7 ± 18.1	4.1 ± 8.4	**<.001**	**<.001**
Mean SpO_2_ (%)	90.9 ± 5.7	94.9 ± 1.9	**<.001**	93.1 ± 4.6	95.3 ± 2.9	**.002**	94.9 ± 3.9	96.1 ± 3.1	**.038**	91.3 ± 5.1	95.0 ± 2.5	**.001**	.287
Nadir SpO_2_ (%)	70.7 ± 14.8	81.3 ± 10.4	**<.001**	73.6 ± 13.9	84.2 ± 9.8	**<.001**	81.3 ± 10.7	85.5 ± 8.2	**.004**	69.3 ± 15.5	79.8 ± 11.7	**<.001**	**<.001**
Laboratory tests													
RBC count (×10^12^/l)	4.81 ± 0.55	4.52 ± 0.47	.289	4.79 ± 0.52	4.74 ± 0.58	.271	4.82 ± 0.56	4.70 ± 0.73	.398	4.79 ± 0.44	4.92 ± 0.49	.356	.541
TG (mmol/L)	2.2 ± 1.5	1.2 ± 0.6	**<.001**	2.1 ± 1.3	1.1 ± 0.7	**<.001**	1.9 ± 1.2	1.0 ± 0.8	**.002**	2.3 ± 1.4	1.1 ± 0.5	**<.001**	.374
TC (mmol/l)	5.0 ± 1.2	4.2 ± 0.8	**<.001**	4.9 ± 1.3	4.0 ± 0.7	**<.001**	4.7 ± 1.1	3.9 ± 0.8	**<.001**	5.1 ± 1.3	4.1 ± 0.7	**<.001**	.402
FPG (mmol/l)	5.9 ± 2.0	4.2 ± 0.6	**<.001**	5.8 ± 2.1	4.1 ± 0.6	**<.001**	5.6 ± 1.8	4.2 ± 0.8	**.001**	6.0 ± 2.1	4.1 ± 0.5	**<.001**	.398
HbA1c (%)	6.4 ± 1.5	5.1 ± 0.5	**<.001**	6.2 ± 1.3	4.9 ± 0.7	**<.001**	6.1 ± 1.2	4.9 ± 0.9	**<.001**	6.5 ± 1.6	5.0 ± 0.4	**<.001**	.421
Blood pressure													
SBP (mmHg)	141.8 ± 17.8	126.0 ± 17.1	**<.001**	138.5 ± 16.9	124.8 ± 15.7	**<.001**	135.2 ± 15.6	122.5 ± 14.9	**.003**	143.9 ± 18.2	125.5 ± 16.5	**<.001**	.263
DBP (mmHg)	85.5 ± 11.8	76.7 ± 12.9	**<.001**	83.2 ± 10.9	75.4 ± 11.2	**<.001**	81.8 ± 10.3	74.6 ± 10.7	**.012**	86.1 ± 12.0	77.2 ± 11.5	**<.001**	.318

^a^Independent samples *t* test within groups. ^b^One-way analysis of variance on the differences between preoperative and postoperative values among the 3 groups: the OHS group, the eucapnic no/mild OSA group, and the eucapnic moderate/severe OSA group. Statistically significant values are indicated in bold. AHI = apnea-hypopnea index, BMI = body mass index, cLac = concentration of lactic acid, DBP = diastolic blood pressure, FPG = fasting plasma glucose, HbA1c = glycated hemoglobin, HCO_3_^−^ = calculated bicarbonate from the arterial blood gas, LAT = longest apnea time, MBS = metabolic and bariatric surgery, ODI = oxygen desaturation index, OHS = obesity hypoventilation syndrome, OSA = obstructive sleep apnea, PaCO_2_ = partial pressure of carbon dioxide in arterial blood, PaO_2_ = partial pressure of arterial oxygen, SBP = systolic blood pressure, SIT90 = the percentage of oxygen saturation less than 90% in total recording time, SpO_2_ = saturation of percutaneous oxygen, TC = total cholesterol, TG = triglycerides.

**Table 4:** Comparison of preoperative characteristics and effectiveness of MBS in the OHS cohort.

	No Resolution (n = 46)	Resolution (n = 105)	*P*
Demographic characteristics			
Age (years)	33.1 ± 10.1	32.4 ± 11.0	.970
Sex (male/female)	30/16	62/43	.593
Anthropometric parameters			
BMI (kg/m^2^)	39.7 ± 6.3	38.6 ± 7.8	.145
ΔBMI (kg/m^2^)	7.2 ± 4.9	11.4 ± 6.3	**<.001**
%TWL	18.9 ± 12.6	28.2 ± 11.0	**<.001**
Surgical procedures			
LSG/LRYGB/SADI-S	30/14/2	58/36/11	.353
Circumference			
Neck (cm)	44.5 ± 4.0	43.9 ± 5.6	.460
ΔNeck (cm)	4.1 ± 5.4	5.7 ± 3.9	.287
Chest (cm)	123.1 ± 16.5	120.4 ± 11.8	**.020**
ΔChest (cm)	10.3 ± 8.2	18.2 ± 15.6	**<.001**
Waist (cm)	123.2 ± 17.6	118.3 ± 13.8	**.013**
ΔWaist (cm)	13.5 ± 15.7	25.1 ± 13.5	**<.001**
Hip (cm)	125.9 ± 15.5	120.3 ± 11.2	**.028**
ΔHip (cm)	10.6 ± 9.4	19.2 ± 13.1	**<.001**
Arterial blood gas			
pH	7.33 ± 0.04	7.34 ± 0.03	.582
ΔpH	0.02 ± 0.05	0.03 ± 0.04	**.021**
PaCO_2_ (mmHg)	49.0 ± 3.1	48.1 ± 2.5	**<.001**
ΔPaCO_2_ (mmHg)	4.4 ± 4.1	5.6 ± 4.2	**<.001**
PaO_2_ (mmHg)	88.7 ± 18.3	91.9 ± 20.6	.834
ΔPaO_2_ (mmHg)	7.6 ± 30.8	13.3 ± 29.1	**<.001**
Portable sleep study			
AHI (events/h)	46.2 ± 32.1	42.7 ± 37.0	.484
ΔAHI (events/h)	26.9 ± 27.6	30.3 ± 34.4	.920
Blood pressure			
SBP (mmHg)	147.2 ± 15.9	139.7 ± 18.1	.095
ΔSBP (mmHg)	14.2 ± 20.3	17.1 ± 17.7	.357
DBP (mmHg)	86.9 ± 11.4	85.0 ± 12.1	.580
ΔDBP (mmHg)	8.0 ± 12.5	9.2 ± 15.9	.961

Statistically significant values are indicated in bold. AHI = apnea-hypopnea index, BMI = body mass index, DBP = diastolic blood pressure, LRYGB = laparoscopic Roux-en-Y gastric bypass, LSG = laparoscopic sleeve gastrectomy, MBS = metabolic and bariatric surgery, OHS = obesity hypoventilation syndrome, PaCO_2_ = partial pressure of carbon dioxide in arterial blood, PaO_2_ = partial pressure of arterial oxygen, SADI-S = single anastomosis duodeno-ileal bypass with sleeve gastrectomy, SBP = systolic blood pressure, %TWL = percentage of total weight loss.

The portable sleep study results demonstrated a substantial reduction in AHI (from 45.1 ± 35.5 events/h to 17.7 ± 18.1 events/h, *P* < .001) and oxygen desaturation index (from 45.5 ± 34.7/h to 14.6 ± 15.5/h, *P* < .001), along with an increase in both mean saturation of percutaneous oxygen saturation (SpO_2_) and nadir SpO_2_.

There were significant reductions in postoperative neck, chest, waist, and hip circumferences. Additionally, postoperative levels of triglycerides, total cholesterol, fasting plasma glucose, and glycated hemoglobin significantly decreased, and hypertension resolution was observed. Red blood cell counts remained comparable between the preoperative and postoperative phases.

### Comparison between patients with and without OHS

We compared outcomes between patients with and without OHS (**Table 3**). In terms of weight loss following MBS, all non-OHS subgroups experienced significant and similar reductions in body weight, with %TWL of 24.8% ± 12.1% in the overall non-OHS group, 24.3% ± 11.8% in the eucapnic no/mild OSA group, and 25.9% ± 12.6% in the eucapnic moderate/severe OSA group, without any significant group differences (*P =* .598). Similar trends of improvement were observed across anthropometric parameters (neck, chest, waist, and hip circumferences), metabolic laboratory indicators, and blood pressure, similar to the changes seen in the OHS group. Analysis of variance of pre-post differences comparing the OHS group, eucapnic no/mild OSA group, and eucapnic moderate/severe OSA group revealed no statistically significant group differences in weight loss outcomes.

Non-OHS patients with moderate-to-severe OSA showed a notable improvement in PaO_2_. Other parameters in the non-OHS group did not exhibit statistically significant pre–post differences. Further intergroup comparisons between OHS, eucapnic no/mild OSA, and eucapnic moderate/severe OSA groups demonstrated statistically significant differences in the pre–post changes of pH, PaCO_2_, PaO_2_, and concentration of lactic acid (**Table 3**).

For sleep study parameters, a similar trend of improvement was observed between the OHS and non-OHS groups. As expected, the degree of improvement in sleep study parameters such as AHI, oxygen desaturation index, percentage of oxygen saturation less than 90% in total recording time, and nadir SpO_2_ was greater in patients with OHS and eucapnic moderate/severe OSA compared to the eucapnic no/mild OSA group.

### Comparison of OHS resolution and nonresolution groups

Notably, in 46 patients (30.5%) hypercapnia either did not resolve at 1-year follow-up (n = 43) or the resolution of hypercapnia could have been partially explained by the recent use of PAP therapy (n = 3). **Table 4** compares the groups with and without OHS resolution. Whereas baseline BMI was similar between the groups (38.6 ± 7.8 kg/m^2^ vs 39.7 ± 6.3 kg/m^2^, *P =* .145), both BMI reduction and %TWL were significantly greater in the resolution group (change in BMI: 11.4 ± 6.3 kg/m^2^ vs 7.2 ± 4.9 kg/m^2^, *P* < .001; %TWL: 28.2 ± 11.0% vs 18.9 ± 12.6%, *P* < .001).

In terms of anthropometric measurements, both groups had similar preoperative neck circumferences and reductions in neck circumference postoperatively. However, the OHS resolution group had smaller baseline chest, waist, and hip circumferences with greater postsurgery reductions in these parameters.

Regarding ABG parameters, the nonresolution group had higher baseline PaCO_2_ levels (49.0 ± 3.1 mmHg vs 48.1 ± 2.5 mmHg, *P* < .001), whereas the baseline pH and PaO_2_ levels were comparable between groups (pH: 7.33 ± 0.04 vs 7.34 ± 0.03, *P =* .582; PaO_2_: 88.7 ± 18.3 mmHg vs 91.9 ± 20.6 mmHg, *P =* .834). Postoperatively, the resolution group showed greater improvements in pH, PaCO_2_, and PaO_2_ compared to the nonresolution group.

No significant differences were found between the groups regarding surgical procedures, portable sleep study results, or blood pressure. Baseline AHI, as well as both systolic and diastolic blood pressures, were similar between groups, and postoperative improvements were comparable.

### Correlation between MBS and hypercapnia resolution

**Figure 3:**
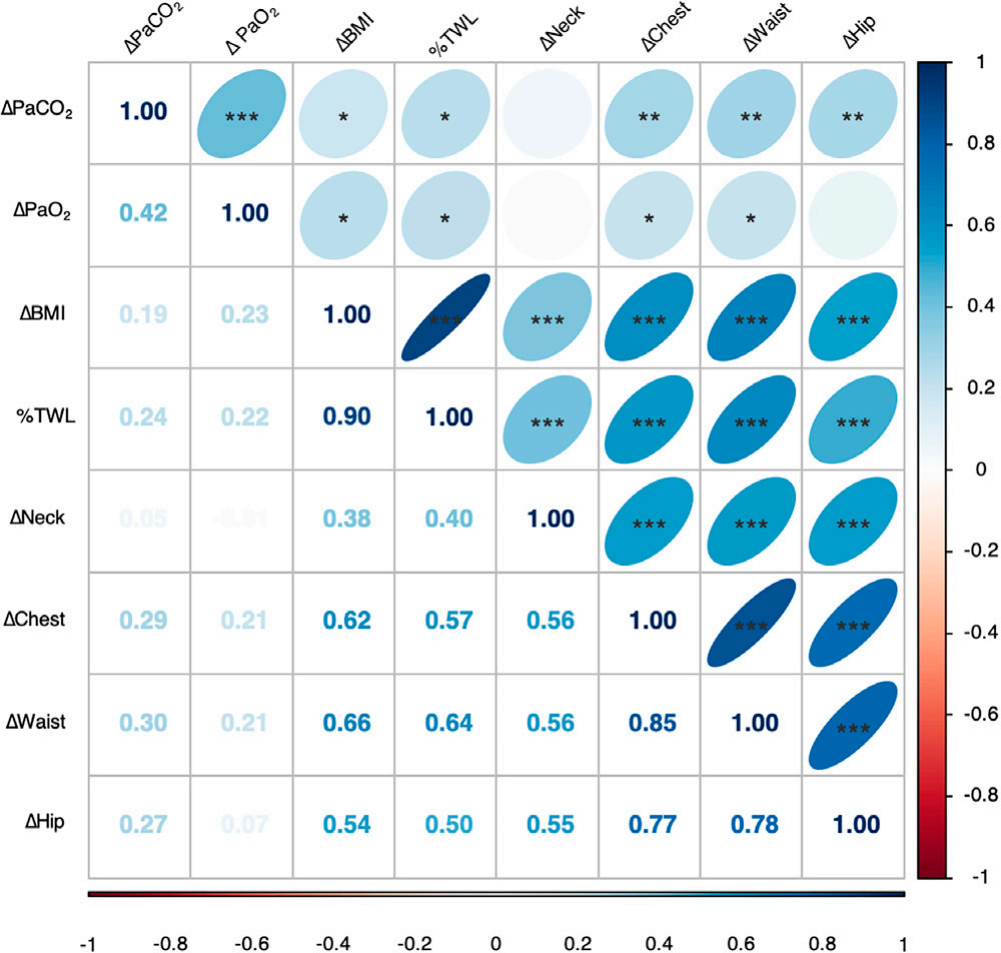
Heatmap of the correlation between covariates of MBS and hypercapnia resolution. The circles represent the Pearson correlation coefficient (*r*) values. Red and blue indicate positive and negative values, respectively. Δ represents the absolute value of the difference between preoperative and postoperative measurements. *P < .05, **P < .01, and ***P < .001. BMI = body mass index, MBS = metabolic and bariatric surgery, PaCO_2_ = partial pressure of carbon dioxide in arterial blood, PaO_2_ = partial pressure of arterial oxygen, %TWL = percentage of total weight loss.

OHS resolution was significantly correlated with weight loss following MBS. As depicted in **Figure 3**, although the correlations were statistically significant, the relationship between change in PaCO_2_ (ΔPaCO_2_) and change in BMI (*r* = .19, *P =* .023) and %TWL (*r =* .24, *P =* .014) showed a weak linear pattern. Additionally, ΔPaCO_2_ showed significant but weak linear correlations with reductions in chest circumference (Δchest, *r =* .29, *P =* .008), waist circumference (Δwaist, *r =* .30, *P =* .006), and hip circumference (Δhip, *r =* .27, *P =* .005). A moderate linear relationship was observed between ΔPaCO_2_ and increase in PaO_2_ (*r =* .42, *P =* .001).

**Figure 4:**
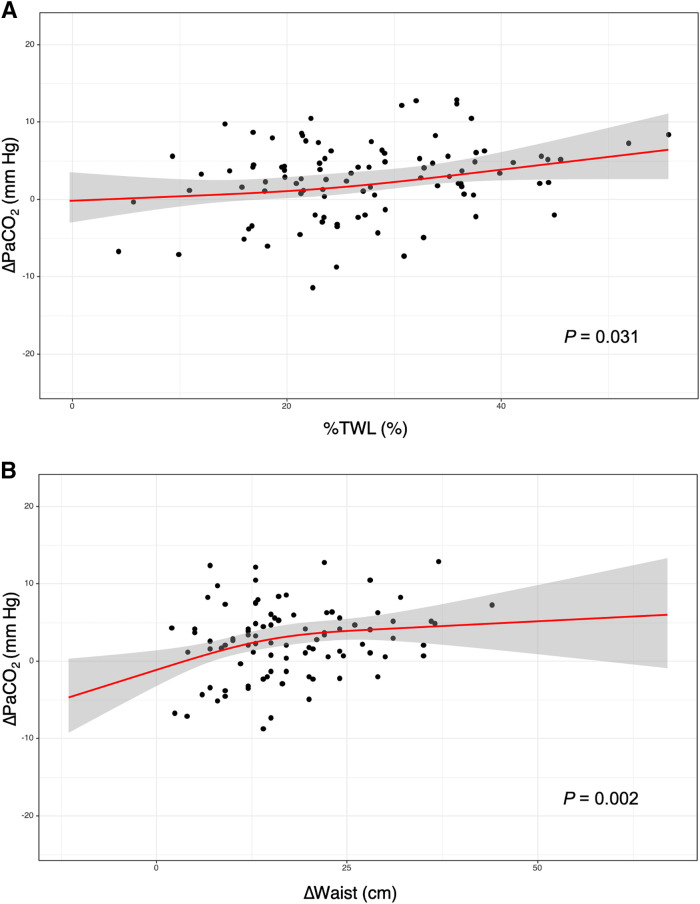
Correlation between MBS covariates and hypercapnia resolution. **(A)** Restricted cubic splines of ΔPaCO_2_ and %TWL. **(B)** Restricted cubic splines of ΔPaCO_2_ and ΔWaist. Points indicate a single data point. Thick lines (with shadows) are modeled data (and 95% confidence interval). Δ represents the absolute value of the difference between preoperative and postoperative measurements. MBS = metabolic and bariatric surgery, PaCO_2_ = partial pressure of carbon dioxide in arterial blood, %TWL = percentage of total weight loss.

Restricted cubic splines analysis demonstrated a dose–response nonlinear relationship between weight loss and OHS resolution. As illustrated in **Figure 4A**, when %TWL was less than approximately 20%, ΔPaCO_2_ remained relatively unchanged. When %TWL exceeded 20%, the slope of ΔPaCO_2_ increased compared to the previous range. Similarly, reduction in waist circumference was associated with notable improvements in PaCO_2_ (**Figure 4B**). However, when waist circumference reduction reached around 25 cm, the rate of improvement in PaCO_2_ reached a plateaue.

### Risk factors for OHS nonresolution after MBS

Univariate and multivariate analyses were performed to identify the risk factors associated with unresolved OHS after MBS (**Table 5**). Multivariate analysis revealed that preoperative pH < 7.35 (odds ratio: 3.921, 95% confidence interval: 2.305–9.140, *P* < .001) and waist circumference (odds ratio: 1.046, 95% confidence interval: 1.031–1.118, *P =* .025) were independent risk factors for unresolved OHS. Conversely, %TWL (odds ratio: 0.917, 95% confidence interval: 0.846–0.965, *P =* .001) was independently associated with OHS resolution.

**Table 5:** Risk factors associated with unresolved OHS after MBS.

	Univariate			Multivariate		
	OR	95% CI	*P*	OR	95% CI	*P*
Demographic						
Age (years)	1.541	1.034–1.969	**.002**	1.063	0.864–1.335	.824
Sex						
Female	Ref					
Male	1.310	0.449–1.644	.272			
Anthropometric parameters						
Weight (kg)	0.989	0.709–1.017	.706			
BMI (kg/m^2^)	1.042	0.986–1.101	.147			
TWL (%)	0.828	0.797–0.912	**<.001**	0.917	0.846–0.965	**.001**
Circumference						
Neck (cm)	0.975	0.911–1.043	.458			
Chest (cm)	1.015	0.988–1.042	.287			
Waist (cm)	1.133	1.004–1.563	**.007**	1.046	1.031–1.118	**.025**
Hip (cm)	1.019	0.996–1.042	.115			
Surgical procedures						
LSG	Ref					
LRYGB	0.926	0.444–1.935	.829			
SADI-S	2.250	0.461–6.957	.316			
Arterial blood gas						
pH						
≥7.35	Ref					
<7.35	5.092	2.593–10.316	**<.001**	3.921	2.305–9.140	**<.001**
PaCO_2_	1.309	0.995–1.551	.172			
PaO_2_ (mmHg)	0.998	0.980–1.017	.833			
HCO3^−^ (mmol/l)	1.042	0.895–1.213	.597			
cLac (mmol/l)	1.138	0.776–1.698	.521			
Portable sleep study						
AHI (events/h)	0.996	0.985–1.007	.482			
ODI (/h)	0.995	0.983–1.006	.360			
LAT (seconds)	1.003	0.937–1.022	.498			
SIT90 (%)	1.001	0.996–1.004	.893			
Mean SpO_2_ (%)	0.961	0.888–1.040	.323			
Nadir SpO_2_ (%)	0.983	0.956–1.012	.250			
SBP						
≤140 (mmHg)	Ref					
>140 (mmHg)	1.355	0.970–1.741	.627			

Statistically significant values are indicated in bold. AHI = apnea-hypopnea index, BMI = body mass index, CI = confidence interval, cLac = concentration of lactic acid, HCO_3_^−^ = calculated bicarbonate from the arterial blood gas, LAT = longest apnea time, LRYGB = laparoscopic Roux-en-Y gastric bypass, LSG = laparoscopic sleeve gastrectomy, ODI = oxygen desaturation index, OR = odds ratio, PaCO_2_ = partial pressure of carbon dioxide in arterial blood, PaO_2_ = partial pressure of arterial oxygen, Ref = reference, SADI-S = single anastomosis duodeno-ileal bypass with sleeve gastrectomy, SBP = systolic blood pressure, SIT90 = the percentage of oxygen saturation less than 90% in total recording time, SpO_2_ = saturation of percutaneous oxygen.

## DISCUSSION

To the best of our knowledge, this retrospective analysis is the largest one exploring MBS in patients with OHS. We demonstrated that MBS is an effective approach for OHS resolution. At 1-year follow-up, 69.5% of patients experienced resolution of OHS despite discontinuation of PAP therapy for 6–9 months. Moreover, these patients experienced a significant improvement in sleep-disordered breathing. Patients who achieved resolution of OHS experienced on average a total weight loss of 28%. Our data also suggest that with a multidisciplinary approach MBS can be safely performed even if patients with OHS or eucapnic moderate-to-severe OSA are not using PAP therapy at home prior to admission to the hospital.

In addition to the perioperative safety and efficacy of MBS in patients with OHS, our study demonstrated a significant improvement in metabolic markers (ie, triglycerides, fasting blood glucose, glycated hemoglobin) and blood pressure in patients undergoing MBS. These findings suggest that, in contrast to PAP therapy, the degree of weight loss achieved with surgical interventions may be an effective means of reducing the cardiometabolic burden of OHS. Although PAP therapy improves symptoms, quality of life, and gas exchange in patients with OHS, weight loss and increase in physical activity are critically important to reduce the cardiometabolic risk in OHS.^6^^,^^10^^,^^11^ To that end, our data suggest that MBS is likely more effective than PAP therapy in reducing future cardiometabolic risk in this patient population.

The American Thoracic Society guidelines^10^ recommend a weight loss target of 25–30% in patients with OHS; however, this was based mostly on expert consensus rather than direct clinical evidence.^10^^,^^11^ Interestingly, our study found that OHS resolution may require %TWL of at least 20% (**Figure 4A**), which aligns closely with the American Thoracic Society guidelines. Insufficient weight loss might not relieve the impeded diaphragm motion caused by redundant adipose tissue surrounding the chest wall and abdominal viscera. The exact amount of weight loss needed for the resolution of OHS, however, remains uncertain and likely varies from patient to patient.^11^ Prior studies have reported that an intensive lifestyle approach with counseling, diet, and exercise leads to a weight reduction of 6–7%, which does not lead to resolution of OHS over the long term.^21^^–^^23^ Another study showed that PaO_2_ increased by 10 mmHg and PaCO_2_ decreased by 3 mmHg after a decrease of the BMI by 13 kg/m^2^.^24^ These results indicate that lower degrees of weight loss do not effectively relieve hypercapnia or hypoxemia, therefore indicating that sufficient weight loss is necessary to achieve OHS resolution.

In terms of surgical procedures, there are differences in the target percentage of weight loss achieved between different types of MBS procedures. However, in the multivariate analysis of this study, the type of surgery was not associated with the resolution of OHS. It is important to note that at our center we did not perform gastric banding. We only performed the types of MBS that lead to larger degree of weight loss such as laparoscopic sleeve gastrectomy, laparoscopic Roux-en-Y gastric bypass, and single anastomosis duodeno-ileal bypass with sleeve gastrectomy.

Multivariate analysis showed that, in addition to weight loss, waist circumference also influenced OHS resolution. Our study found that PaCO_2_ improved significantly as waist circumference decreased, particularly within the first 25 cm of reduction. This aligns with findings from Harada et al^25^ who also noted a correlation between the severity of OHS and waist circumference. Abdominal obesity can lead to a restrictive respiratory impairment, increasing the risk of hypoxemia and hypercapnia.^26^^–^^28^ Prior studies^29^^–^^32^ have linked central fat distribution to greater respiratory decline than peripheral obesity. The association between central fat distribution and gas exchange has been demonstrated to be stronger than that with weight or BMI. It is worth noting that our study also revealed that after waist circumference reduction reached a certain threshold, the improvement in PaCO_2_ reached a plateau. However, it is important to consider racial and ethnic differences in fat distribution. In our study, the average BMI of the OHS group was 39.1 ± 6.8 kg/m^2^, which differs from studies performed in Western countries that have reported more severe obesity (BMI ≥ 40 kg/m^2^).^6^ It has been reported that Asians are more prone to developing visceral rather than peripheral adiposity,^20^^,^^33^ which may explain the relevance of waist circumference as a consistent marker across different Asian populations. Moreover, it is important to point out that the definition of class 3/severe obesity is different in Asian populations compared to Western countries.^34^ A BMI ≥ 37.5 kg/m^2^ is considered severe obesity in China.^35^ Central adiposity is more strongly associated with metabolic disorders and an increased risk of severe comorbidities.^36^ These variations could lead to differences in the cutoff values used to define risk, potentially introducing heterogeneity in future studies, underscoring the need for further investigation into how these factors may vary across populations and regions.

Finally, we observed that a preoperative ABG pH below 7.35 was associated with lower OHS resolution. This likely reflects preoperative decompensation, because a pH < 7.35 suggests a more severe acid–base imbalance and may indicate a greater degree of OHS severity.

In clinical practice, preoperative use of PAP therapy is considered helpful in optimizing perioperative stability in patients with OHS, although no consensus currently exists regarding its optimal duration or treatment targets prior to MBS. In our study, all 151 patients in the OHS cohort received inpatient preoperative PAP therapy after the diagnosis OHS was confirmed by the MDT. Despite relatively short-term PAP use before surgery (6.3 ± 1.8 days), no serious postoperative cardiopulmonary complications occurred, indicating that short-term PAP therapy may be adequate to support perioperative stability and allow patients to safely undergo surgery that may offer the potential for resolution of OHS. However, further research is needed to determine the optimal PAP therapy duration, modality, treatment targets, and other parameters required to guide surgical readiness.

Our study does have limitations. First, only MBS candidates were included, which means most patients were young and had fewer cardiopulmonary comorbidities and a shorter metabolic disorder history, potentially limiting the generalizability of our findings. Second, as a retrospective study, certain data were inherently difficult to obtain, such as postoperative PAP usage and adherence, which limits our ability to evaluate the impact of these factors on outcomes. Third, approximately 20% of patients failed to follow up at the 1-year mark, which is consistent with the challenges faced by other tertiary hospitals in China, where patients often come from geographically distant areas, making long-term follow-up more challenging. Finally, our analysis focused on 1-year outcomes post-MBS, and long-term studies are needed to confirm the durability of these results.

## CONCLUSIONS

This study demonstrated that MBS is an effective and safe intervention for improving OHS, with 69.5% of patients achieving resolution at 1-year follow-up. The most important predictor of OHS resolution was sufficient weight loss, particularly reductions in waist circumference, which were associated with improvements in gas exchange. Acidic preoperative pH and higher degree of central obesity were identified as key risk factors for not achieving resolution of OHS. Our findings suggest that weight loss of at least 20% is necessary for significant clinical improvement and that MBS should be considered a viable treatment option for patients with OHS.

## DISCLOSURE STATEMENT

All authors have seen and approved the manuscript. This research was supported by National Natural Science Foundation of China (grant no. 82070917), Clinical Research Program of 9th People’s Hospital affiliated to Shanghai Jiao Tong University School of Medicine (JYLJ202216), and the Project of Biobank (YBKB202219) from Shanghai Ninth People’s Hospital, Shanghai Jiao Tong University School of Medicine. The authors report no conflicts of interest.

## OPEN ACCESS

Copyright 2025 The Authors. This is an open access article, distributed under the Creative Commons Attribution 4.0 International License. Sharing and adaptation are permitted provided attribution to its original publication in the Journal of Clinical Sleep Medicine is made in accordance with the license.

## Supplemental Materials

10.5664/jcsm.11750 Supplemental Materials

## ABBREVIATIONS

ABG: arterial blood gas
AHI: apnea-hypopnea index
BMI: body mass index
MBS: metabolic and bariatric surgery
MDT: multidisciplinary team
OHS: obesity hypoventilation syndrome
OSA: obstructive sleep apnea
PaCO_2_: partial pressure of carbon dioxide in arterial blood
PAP: positive airway pressure
%TWL: percentage of total weight loss
