# Supplementary material for: Impact of bariatric surgery on the resolution of obesity hypoventilation syndrome at 1-year follow-up: a retrospective study
Source: J Clin Sleep Med. 2025 Oct 1;21(10):1665–78. doi: 10.5664/jcsm.11750 (PMC12493080; doi:10.5664/jcsm.11750)
Supplement: Supplementary file 1 [file jcsm.11750.sm001.pdf]

The measurement methods for neck, chest, waist, and hip circumference

- **Neck Circumference**: Measured in a standing posture using a tape measure around the midpoint of the neck.
- **Chest Circumference**: Measured at the level of the nipples in a horizontal plane during both normal inspiration and expiration.
- **Waist Circumference**: Measured with a tape measure at the midpoint between the iliac crest and the lower rib.
- **Hip Circumference**: Measured at the widest part of the hips using a horizontal tape measure.

Reference:

Preedy VR, ed. Handbook of Anthropometry: Physical Measures of Human Form in Health and Disease. 1st ed. New York, NY: Springer; 2012. doi:10.1007/978-1-4419-1788-1.

Standard Operating Procedure (SOP) for Perioperative Management of Obesity Hypoventilation Syndrome (OHS)

Purpose

This is a structured multidisciplinary team (MDT) approach for the perioperative management of elective bariatric patients with Obesity Hypoventilation Syndrome (OHS). Individual patient care should be tailored based on MDT judgment and specific patient needs. The purpose of this diagnosing and treatment framework is to minimize complications, optimize outcomes, and ensure patient safety during bariatric perioperative periods. This standard operating procedure (SOP) applies to all healthcare providers to ensure consistency and safety in care delivery.

The MDT Composition an The Scope of MDT Work

OHS is a complex condition that requires a MDT approach for effective management. The MDT typically includes healthcare professionals from various specialties who collaborate to provide comprehensive care.

The key responsibilities of the MDT in managing OHS in our hospital are as follow:

1. Respiratory Physicians/Pulmonologists

Diagnosis: Confirm the diagnosis of OHS through clinical evaluation, blood gas analysis, and sleep studies.

Management: Initiate and oversee non-invasive positive pressure ventilation (NIPPV) or positive airway pressure (PAP) therapy.

Monitoring: Regularly assess the patient's respiratory function and adjust treatment if necessary.

2. Sleep Specialists

Sleep Studies: Conduct and interpret sleep study to diagnose sleep-disordered breathing, such as obstructive sleep apnea (OSA) and OHS.

Therapy Optimization: Optimize continuous positive airway pressure (CPAP) or bilevel positive airway pressure (BPAP) settings based on sleep study results.

3.Cardiologists

Cardiac Assessment: Evaluate and manage cardiovascular comorbidities such as hypertension, heart failure, or pulmonary hypertension.

Monitoring: Regularly monitor cardiac function, especially in patients with severe OHS.

4.Endocrinologists

Metabolic Assessment: Evaluate and manage comorbid conditions such as diabetes, hyperlipemia and metabolic syndrome.

Medical Therapy: Consider pharmacological interventions for perioperative weight loss if appropriate.

5.Bariatric Surgeons

Surgical Evaluation and screen: Assess the suitability of bariatric surgery for patients with severe obesity who have not achieved adequate weight loss through lifestyle interventions and screening suspicious OHS patients

Personalized Treatment Planning: Tailor the most appropriate surgical procedure based on the patient's health condition, weight loss goals, and lifestyle.

Postoperative Care: Provide follow-up care to monitor weight loss and manage any complications.

6.Anesthesiologists

Preoperative Assessment: Evaluate and optimize patients for surgery, particularly those undergoing bariatric surgery.

Anaesthesia processing: Ensure safety during anesthesia in accordance with specific processing flow.

Postoperative Care: Manage pain and respiratory support in the immediate postoperative period. Determine whether the patient should be transported to surgical intensive care unit (SICU).

7.Psychologists/Psychiatrists

Behavioral Therapy: Address psychological barriers to weight loss, such as emotional eating or depression.

Cognitive Behavioral Therapy (CBT): Provide CBT for insomnia or other sleep-related issues.

8.Pharmacists

Medication Management: Review and manage medications, ensuring there are no contraindications or interactions that could exacerbate OHS.

Education: Educate patients on the proper use of prescribed medications and potential side effects.

9.Dietitians/Nutritionists

Weight Management: Develop personalized dietary plans to promote weight loss, which is a cornerstone of OHS management.

Education: Provide education on healthy eating habits and long-term weight maintenance.

10.Bariatric Nurses

Pre-operative and post-operative care: prepare patients for the realities of surgery, assist during the procedure, and manage post-operative recovery, focusing on wound care, pain management, and prevention of complications. Educating patients about the nutritional adjustments necessary before and after surgery, including portion control, dietary restrictions, and the importance of a balanced diet.

Patient Education: Educate patients on the use of NIPPV devices, recognizing symptoms of worsening condition, and the importance of adherence to therapy.

Coordinating with the surgical team and other healthcare providers: Acting as a liaison between patients and the broader medical team, including surgeons, dietitians, and therapists, to provide cohesive and comprehensive care.

11.SICU Intensivists

Preoperative Assessment:

- ➢ Evaluate respiratory function, including sleep studies and arterial blood gas (ABG).
- ➢ Optimize ventilation strategies, such as NIV or CPAP, to improve baseline respiratory status.
- ➢ Assess comorbidities (e.g., cardiovascular, metabolic) and ensure they are managed appropriately.

Intraoperative Support:

- ➢ Collaborate with anesthesiologists to ensure safe airway management and ventilation during surgery.
- ➢ Monitor for complications like hypoxia, hypercapnia, or hemodynamic instability.

Postoperative Care:

- ➢ Manage ventilation in the SICU, often using NIV or mechanical ventilation, to prevent respiratory failure.
- ➢ Monitor for complications such as atelectasis, pneumonia, or opioid-induced respiratory depression.
- ➢ Gradually transition to spontaneous breathing and ensure adequate oxygenation and ventilation before discharge from the SICU.

12.Physiotherapists

Exercise Programs: Design and supervise exercise programs tailored to the patient’s physical capabilities to improve cardiovascular fitness and promote weight loss.

Breathing Exercises: Teach techniques to improve respiratory muscle strength and endurance.

The MDT approach ensures that all aspects of OHS are addressed, from diagnosis and treatment to long-term management and support. Effective communication and collaboration among team members are crucial for optimizing patient outcomes.

Multidisciplinary Collaborative Diagnosis and Treatment Protocol

1.Preoperative Assessment and Preparation

1.1 Patient Evaluation

- Medical History:
  - Document severity of OHS, including baseline daytime ABG values, pulmonary function tests, and sleep study results.
  - Assess for comorbidities (e.g., obstructive sleep apnea, diabetes, hypertension, cardiovascular disease).
  - Evaluate for signs of right heart failure or pulmonary hypertension.
  - Review previous use of PAP therapy (e.g., CPAP, BPAP).
- Physical Examination:
  - Focus on respiratory, cardiovascular, and neurological systems
  - Assess for signs of right heart failure (e.g., peripheral edema, jugular venous distension).
  - Assess airway for potential difficulty in intubation.
- Diagnostic Tests:
  - ABG: Evaluate baseline $PaCO_2$ and PaO_2 levels.
  - Pulmonary Function Tests (PFTs): Assess lung volumes and mechanics.
  - Echocardiogram: Evaluate for pulmonary hypertension and right ventricular dysfunction.
  - Sleep study (if not previously done) to assess severity of sleep-disordered breathing.

1.2 Risk Stratification

- Classify patients as low or high risk based on OHS severity and comorbidities.

1.3 Preoperative Optimization

- Respiratory Optimization: Initiate or optimize NIPPV (e.g., CPAP or BPAP) to ensure patients are compliant with PAP therapy preoperatively.
- Weight Management: Encourage weight loss if surgery is elective and time permits.
- Comorbidity Management: Optimize control of diabetes, hypertension, and cardiovascular disease. Treat any active infections or exacerbations of chronic conditions.

1.4 Anesthetic & SICU Intensivists Consultation

- Discuss risks of anesthesia (including respiratory failure, prolonged intubation, and postoperative complications), postoperative monitoring and NIPPV support.

2.Intraoperative Management

2.1 Anesthetic Considerations

- Airway Management:
  - Anticipate difficult intubation and have advanced airway equipment available.
  - Use video laryngoscopy if necessary.
- Ventilation Strategy:
  - Use lung-protective ventilation strategies (low tidal volumes, positive end-expiratory pressure (PEEP), and recruitment maneuvers).
  - Monitor end-tidal CO_2 and arterial blood gases to avoid hypercapnia.

- Positioning:
  - Position the patient in a semi-upright or reverse Trendelenburg position to improve ventilation.

2.2 Monitoring

- Continuous monitoring of oxygen saturation (SpO_2), end-tidal CO_2, and hemodynamics.
- Invasive arterial blood pressure monitoring for high-risk patients or prolonged surgeries.

2.3 Fluid Management

- Avoid fluid overload to prevent pulmonary edema.
- Use goal-directed fluid therapy if indicated.

3.Postoperative Management

3.1 Immediate Postoperative Care

- Monitoring:
  - Transfer to a monitored setting (e.g., high-dependency unit or SICU) based on risk stratification and MDT decision. No general observation unit directly, If the diagnosis is OHS.
  - Continuous SpO_2 monitoring, percutaneous carbon dioxide monitoring, and intermittent ABG checks to assess ventilation. Monitor for signs of respiratory failure (e.g., increased work of breathing, hypoxemia, hypercapnia).
- Respiratory Support:
  - Resume NIPPV immediately post-extubation if indicated (e.g., high-risk or OHS patients) to avoid respiratory distress.
  - Ensure proper fit and settings for NIPPV devices.

3.2 Pain Management

- Use multimodal analgesia to minimize opioid use and reduce respiratory depression.
- Consider regional anesthesia techniques (e.g., epidural or nerve blocks) when appropriate.

3.3 Mobilization and Physiotherapy

- Early mobilization to prevent atelectasis and venous thromboembolism (VTE).
- Encourage incentive spirometry and chest physiotherapy.

3.4 Complication Management and Prevention

- Hypoxemia: Administer supplemental oxygen and escalate to NIPPV or mechanical ventilation if necessary.
- Hypercapnia: Adjust PAP settings or initiate NIPPV to improve ventilation.
- Pulmonary Hypertension: Optimize oxygenation and avoid hypoxia/hypercapnia.
- VTE Prophylaxis: Administer pharmacological and mechanical prophylaxis as per guidelines.
- Infection Prevention: Use prophylactic antibiotics if indicated.
- Monitor for signs of surgical site infection or pneumonia.

3.5 Discharge Planning

- Ensure stable respiratory status and adequate pain control before discharge.
- Provide clear instructions for continued use of NIPPV at home.
- Arrange follow-up with pulmonologist, or bariatric specialist and primary care physician.

4. Follow-up

4.1 Follow-up Schedule

- Patients should be followed at 1, 3, 6, and 12 months postoperatively, then annually to assess clinical progress and long-term outcomes.

4.2 Multidisciplinary Follow-up Components

- Cardiovascular and Respiratory Management
  - OHS Symptom Monitoring: Assess symptom relief, including daytime hypersomnolence and dyspnea.
  - NIPPV Therapy: Adjust settings based on $PaCO_2$ levels and assess adherence and determine discontinuation feasibility.
  - Sleep Studies: Repeat if clinically indicated to reassess OSA severity.
  - Cardiovascular Commobidities: Assess pre-commobidities relief (if there was preoperatively).
- Weight loss and Metabolic Monitoring
  - Weight and BMI Tracking: Document %TWL and detect weight regain.
  - Glycemic Control: Monitor for diabetes status.
  - Nutritional Assessment: Identify deficiencies and ensure dietary adequacy.
- Surgical and Lifestyle Monitoring
  - Complication Surveillance: Identify long-term post-surgical issues.
  - Lifestyle and Behavioral Support: Reinforce dietary and physical activity adherence and address psychological barriers to weight maintenance.

5. Documentation and Communication

- Document all assessments, interventions, and patient responses in the medical record, especially the details of PAP therapy settings, intraoperative ventilation parameters, and postoperative respiratory status.
- Communicate and upload the perioperative plan and any changes to the multidisciplinary team and office automation system at any time.

6. Quality Assurance and Improvement

- Regularly review outcomes and complications in OHS patients undergoing bariatric surgery quarterly.
- Update the protocol based on new evidence or institutional experience.
